# Supplementary figures and images for: MetaPro: a scalable and reproducible data processing and analysis pipeline for metatranscriptomic investigation of microbial communities
Source: Microbiome. 2023 Jun 27;11:143. doi: 10.1186/s40168-023-01562-6 (PMC10294448; doi:10.1186/s40168-023-01562-6)

## A) NOD mouse gut

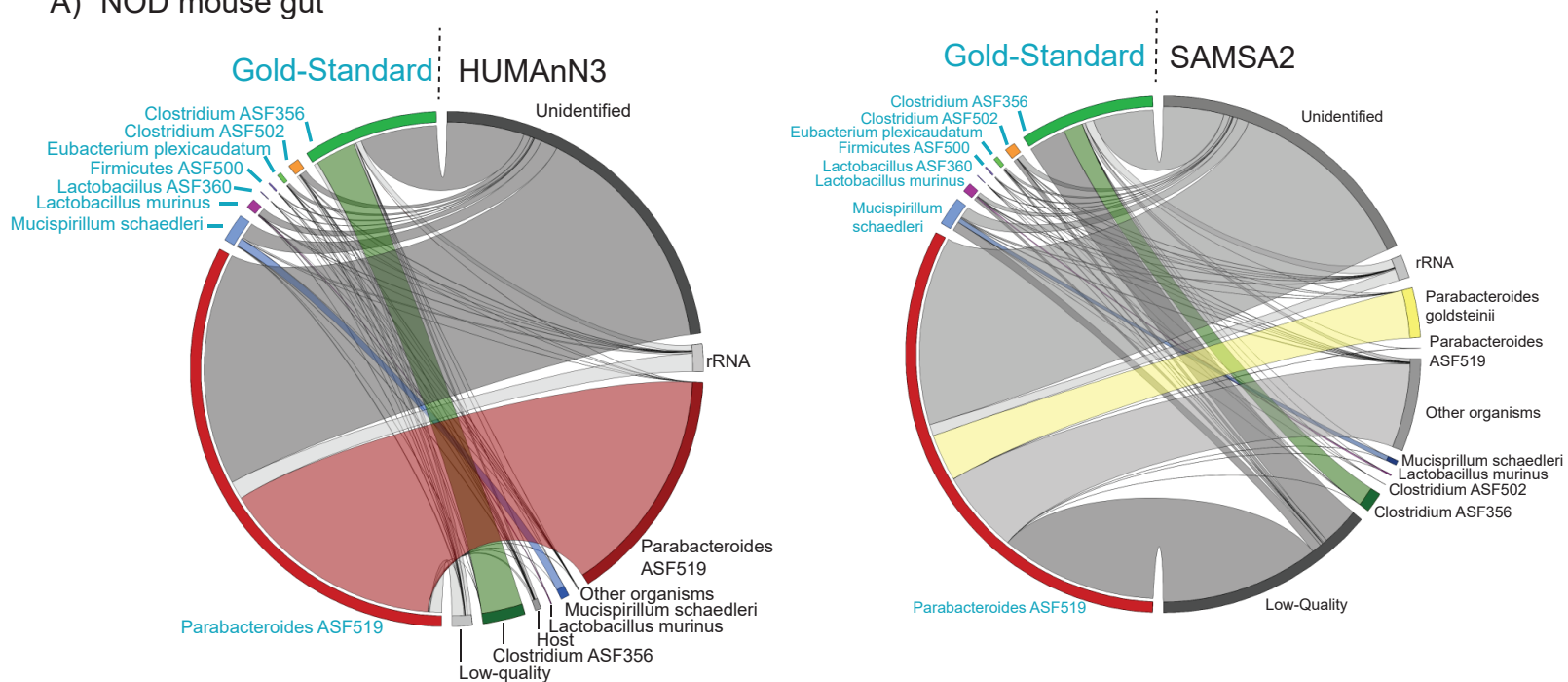

## B) Kimchi

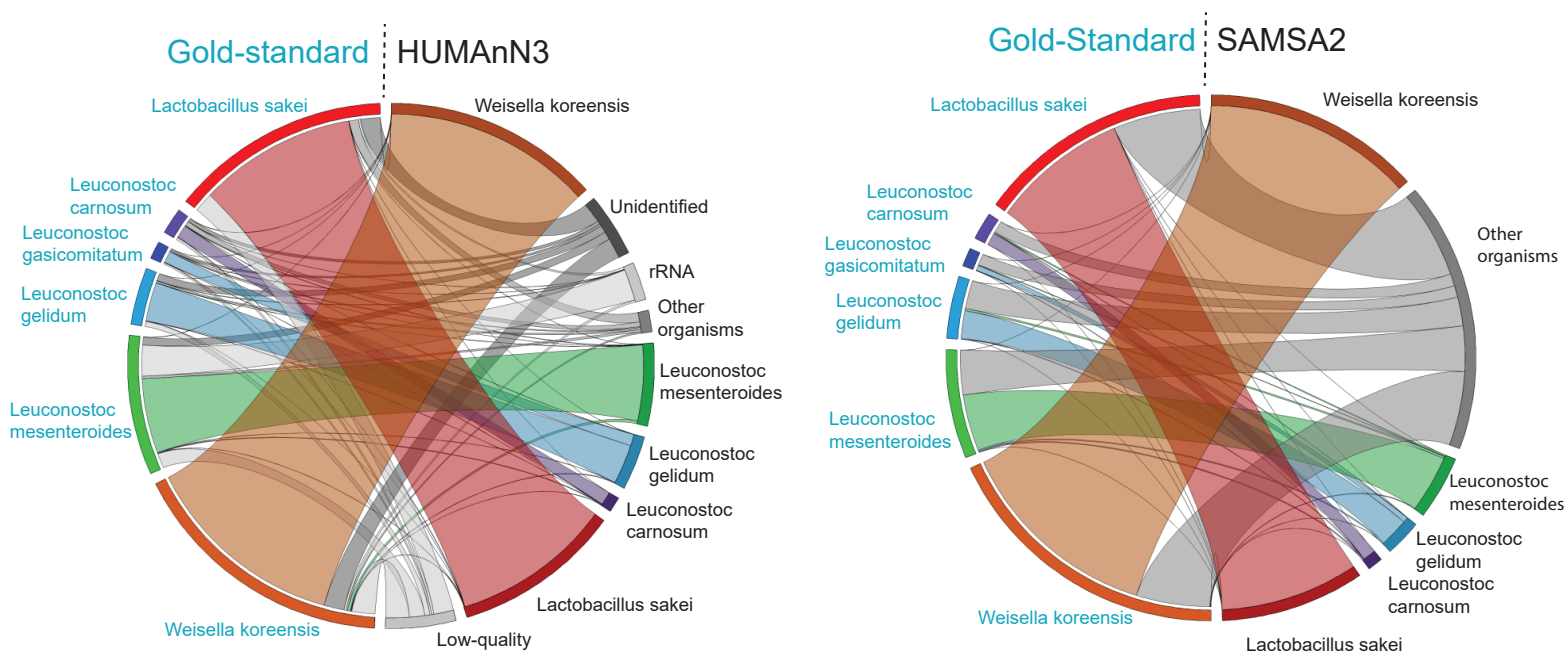

Supplement: Supplementary file 2 — Additional file 1: Fig. S1. Read relationships of HUMAnN3 and SAMSA2 against The Gold standard. Chord diagrams showing the relationships of the gold-standard annotated reads of the NOD mouse gut (A) and kimchi datasets (B) as they are processed by the HUMAnN3 and SAMSA2 pipelines. Each arc of the diagram is a category of reads. Each band joining 2 arcs represents the proportion of reads that map between categories. Many of the unidentified mouse gut reads in HUMAnN3 were identified as Parabacteroides ASF519. Similar to MetaPro, SAMSA2 identified a portion of the Parabacteroides ASF519 gold-standard reads to be Parabacteroides goldsteinii. In the kimchi set, HUMAnN3 has more branching chords than MetaPro. SAMSA2 has a larger proportion of reads in Other organisms compared to MetaPro. In an ideal scenario, there would be a 1:1 relationship between the Gold-standard and the pipelines. [file 40168_2023_1562_MOESM1_ESM.pdf]

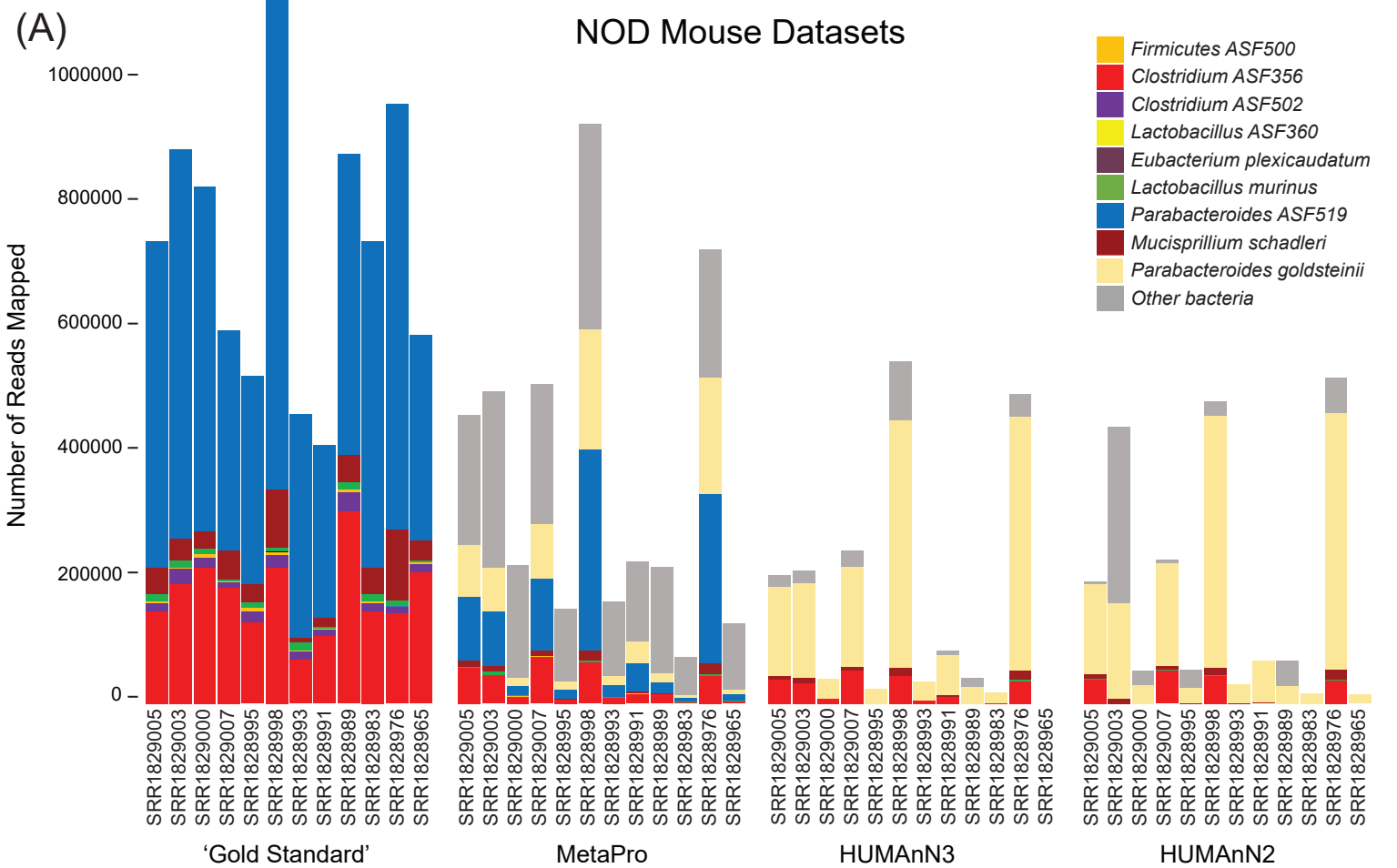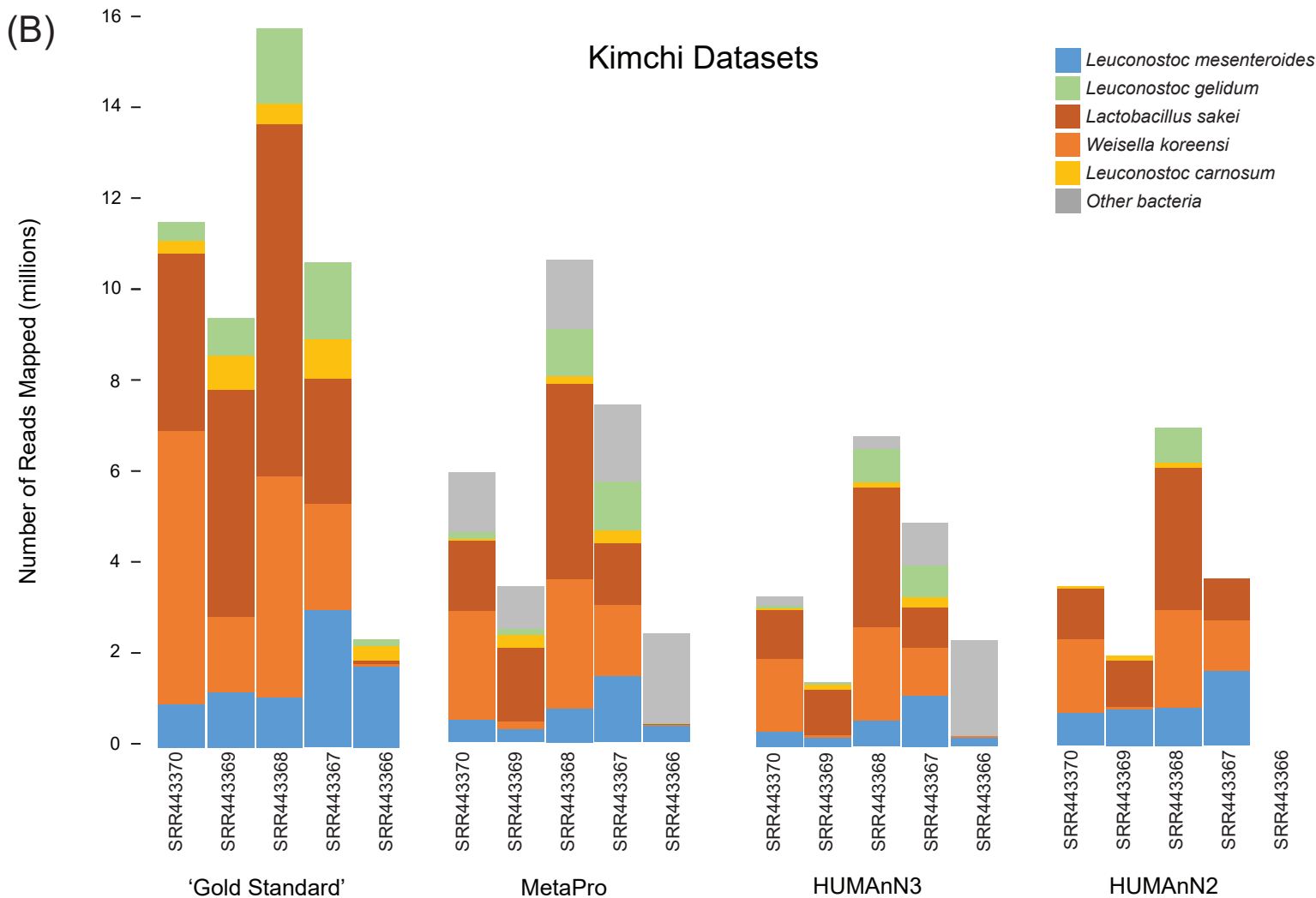

Supplement: Supplementary file 3 — Additional file 2: Fig. S2.Gene annotation performance of MetaPro, HUMAnN3, and HUMAnN2. Stacked barcharts depicting the number of reads annotated to specific taxa in (A) NOD mouse samples, and (B) Kimchi samples by BWA alignments, MetaPro, HUMAnN3, and HUMAnN2. The NOD mouse datasets were generated from gut samples from mice inoculated with a defined microbial consortium (Altered Schaedler Flora (ASF); [29]). In addition to the 8 taxa associated with ASF, reads were also assigned to Parabacteroides goldsteinii, a close relative of Parabacteroides ASF519 (see legend). The kimchi datasets comprise five major taxa (see legend; [30–34]). It should be noted that Leuconostoc gasicomitatum reported in the original publication is currently classified as a subspecies of Leuconostoc gelidum. For NOD sample SRR1828965, HUMAnN3 did not annotate any reads; for kimchi sample SRR443366, HUMAnN2 did not annotate any reads. [file 40168_2023_1562_MOESM2_ESM.pdf]

(A) NOD Mouse Datasets

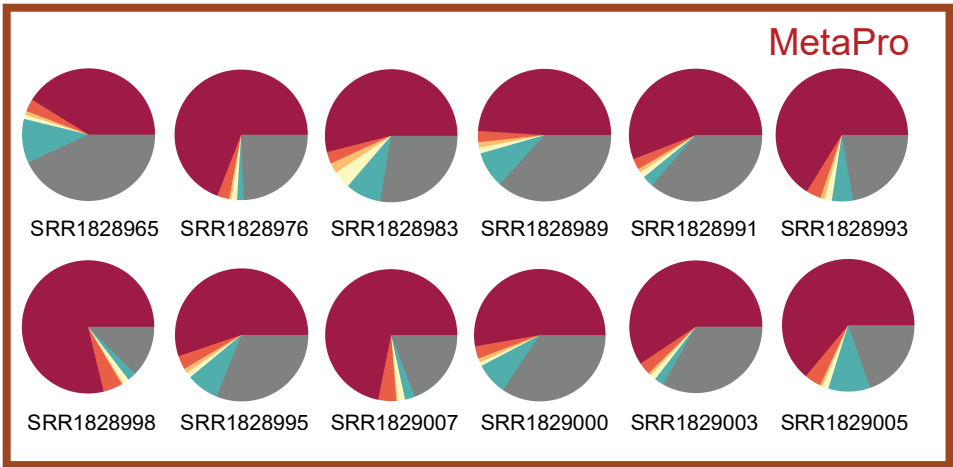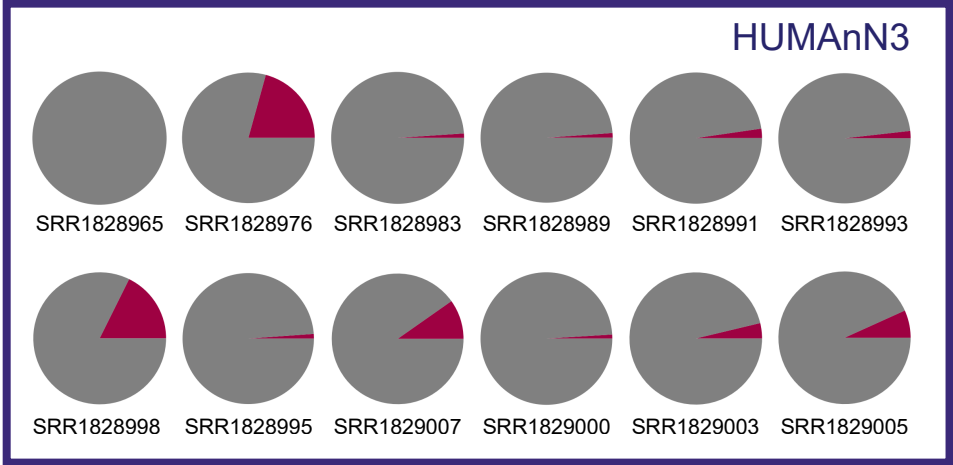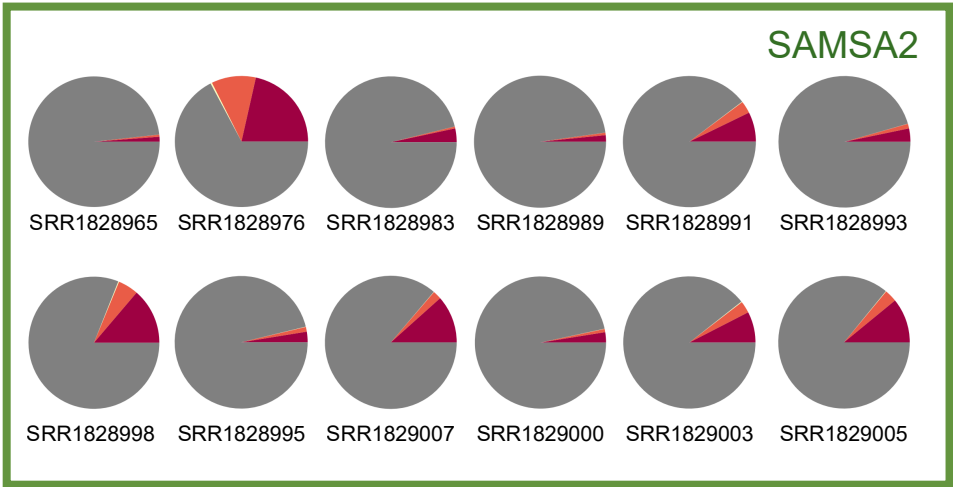

(B) Kimchi Datasets

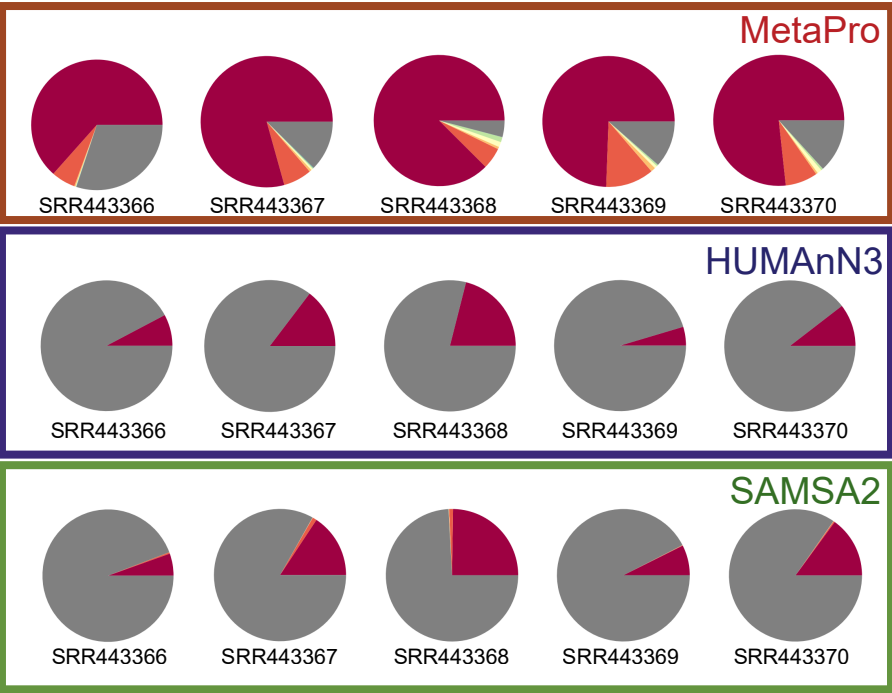

(C) Human Oral Datasets

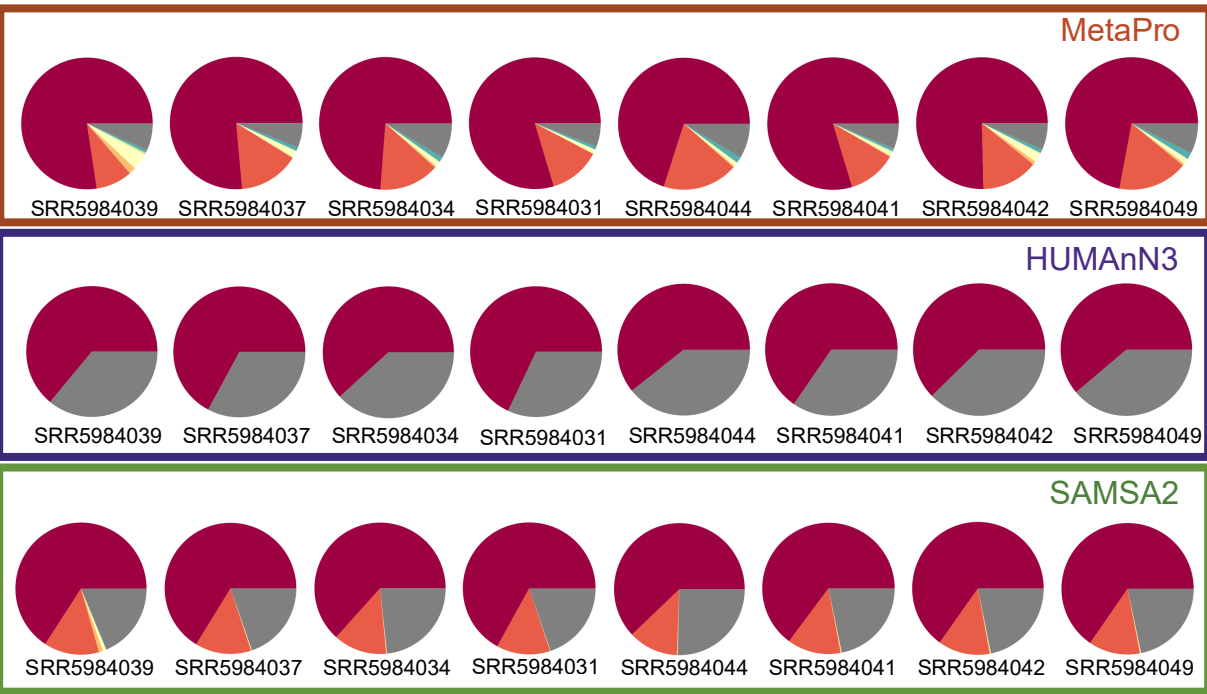

Supplement: Supplementary file 4 — Additional file 3: Fig. 3. Taxonomic classification performance of MetaPro, HUMAnN3, and HUMAnN2. For the NOD mouse (A) and Kimchi (B) datasets, each pie chart shows a breakdown of taxonomic assignments at the different taxonomic levels indicated, that are closest to the last common ancestor of the expected bacteria within the sample. For the human oral datasets (C), given the lack of gold standard assignments, each pie chart represents the relative abundance of reads assigned to different taxonomic levels. Unclassified reads represent annotated reads with no assigned taxon. The graphs indicate a substantial improvement in HUMAnN3’s annotation abilities over HUMAnN2. [file 40168_2023_1562_MOESM3_ESM.pdf]

## (A) NOD Mouse ECs

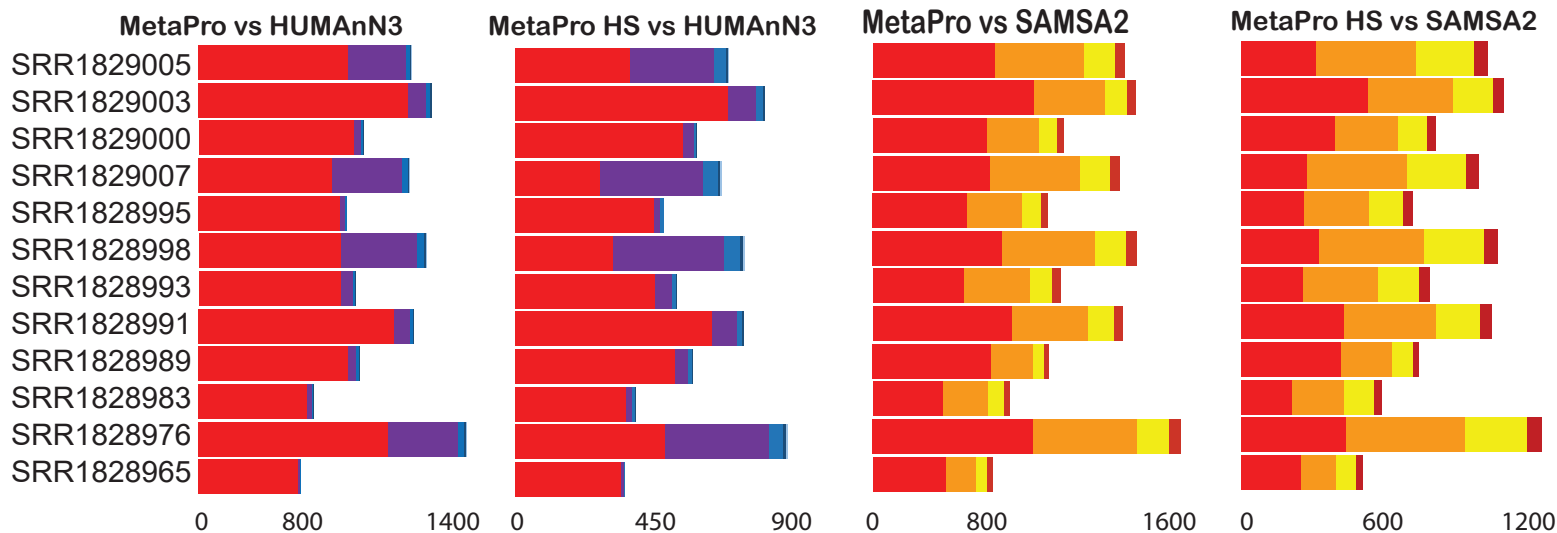

## (B) Kimchi ECs

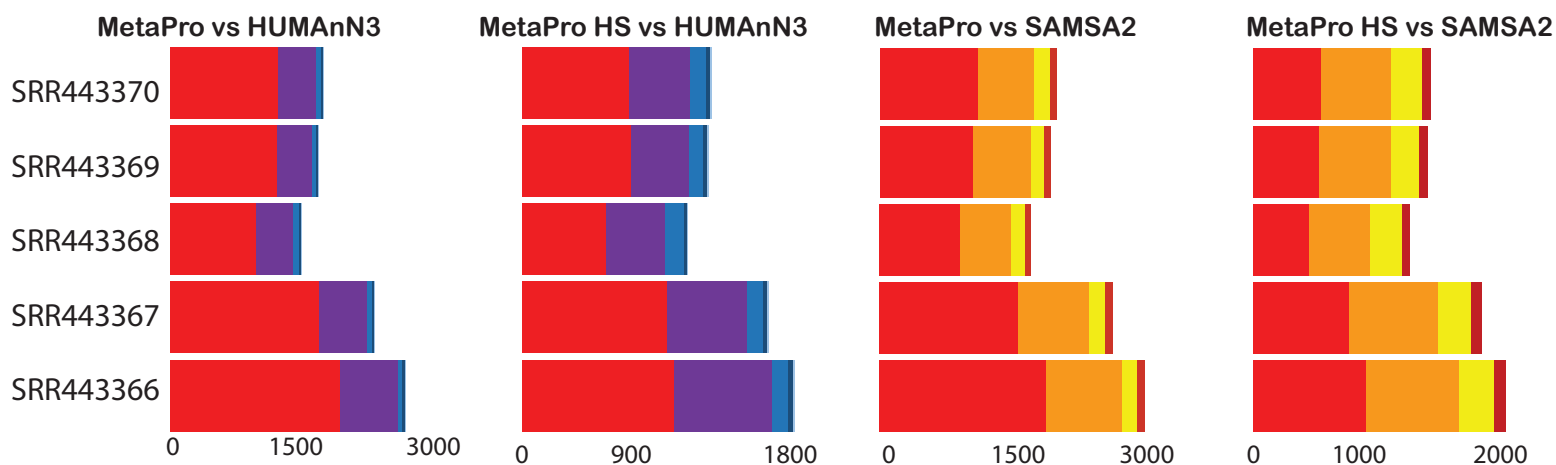

## (C) Human Oral ECs

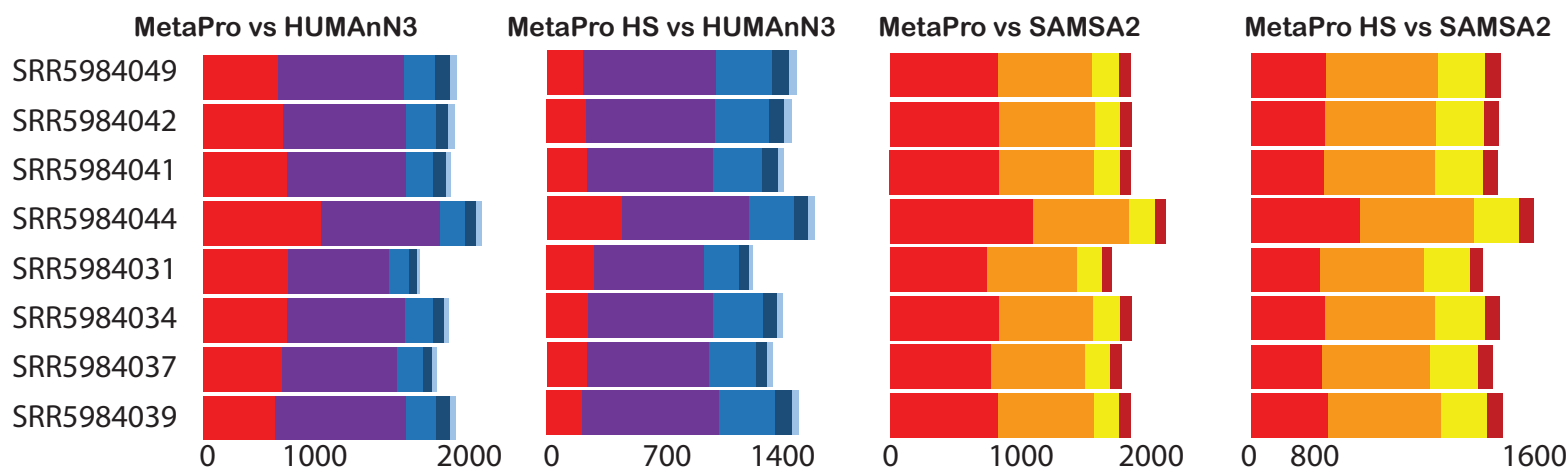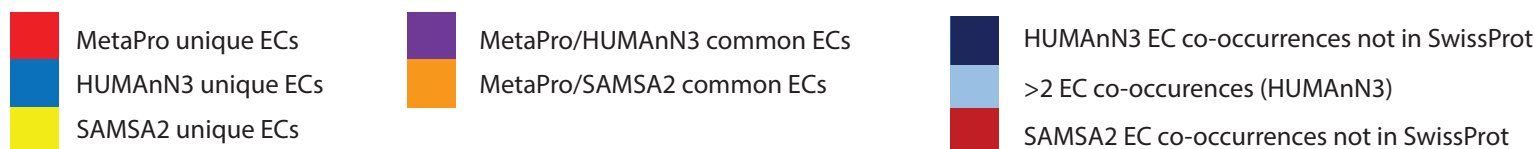

Supplement: Supplementary file 5 — Additional file 4: Fig. S4.Enzyme annotation performance of MetaPro and HUMAnN2. Stacked barcharts indicate the number of enzymes, as defined through enzyme classification (EC) assignments, annotated by each pipeline for the three sets of datasets: (A) NOD mouse, (B) kimchi, and (C) human oral biofilm data. In addition to displaying ECs unique or shared between MetaPro and HUMAnN2, also shown are ECs, predicted by HUMAnN2 to occur in combination with another EC, in the same transcript, with no supporting evidence that such a combination has been previously observed (as defined through Swiss-Prot annotations). Further, for HUMAnN2, we show the number of EC assignments that occur in combinations of three or more ECs. [file 40168_2023_1562_MOESM4_ESM.pdf]
